# Epithelial Cancer of the Lip

**Published:** 1861-05

**Authors:** 


Epithelial Cancer of the Lip.—Mr. Jonathan Hutchinson has given us in recent numbers of the *London Medical Times and Gazette* interesting statistical clinical records of epithelial cancer of the lip. His statistical analysis extends over 127 cases.

Women are the subjects of this disease in the proportion only of 5 to every 100 males ; and when it does occur in women, it is usually in those who have been accustomed to smoke. The lower lip is affected in 90 per cent. of the cases, the angle of the mouth in 6 per cent., and the upper lip in 4

per cent. The average age of patients suffering from cancer of the lip is 58 years, the extremes in the series being 28 and 82.

Operations were performed in all the 127 cases. Although many of them required extensive incisions, and, in three, glands were removed from under the jaw at the same time, only three ended fatally. One, a man aged 62, died on the ninth day of erysipelas of the fauces; one, a man aged 54, died after an extensive operation, "of erysipelas, attended by an eruption like that of scarlet fever;" and the third, a man 59 years of age, died from erysipelas on the seventh day. This rate of mortality Dr. Hutchinson justly considers as scarcely worth noticing when we call to mind the fearful nature of the disease, when not interfered with. The great point is to attend to the general health of the patient and keep him removed from any risk of contagion from erysipelas.

In reference to the liability to a return of the disease in the wound or cicatrix, in 120 cases out of the 127 the wound healed for the time being, and was reported sound when the patient left the hospital. The following table shows the results of the 127 cases:

| | |
|---|---|
| Died within ten days of the operation, | 3 |
| Had return of cancer in the wound, | 4 |
| Had return of cancer in the cicatrix at different periods after the operation, | 9 |
| Had cancerous disease of the lymphatic glands subsequently, | 5 |
| Had cancerous disease of the opposite part or the lip, | 3 |
| No further note than that patients recovered and left the Hospital with sound cicatrices, | 105* |

* In the above classes three cases are mentioned twice on account of their results entitling them to a place in more than one of the groups.

